# Downregulation of hnRNP K by RNAi inhibits growth of human lung carcinoma cells

**DOI:** 10.3892/ol.2014.1832

**Published:** 2014-01-28

**Authors:** FENGMING TANG, WEIMIN LI, YAN CHEN, DONGMEI WANG, JUAN HAN, DAN LIU

**Affiliations:** 1 Department of Respiratory Medicine, West China Hospital of Sichuan University, Chengdu, Sichuan 610041, P.R. China; 2 Department of Respiratory Medicine, The First People’s Hospital of Yibin, Yibin, Sichuan 644000, P.R. China

**Keywords:** heterogeneous nuclear ribonucleoprotein K, siRNA, A549

## Abstract

Lung cancer is one of the most common malignancies worldwide, but its pathogenesis remains unknown. The current study examined the effects of heterogeneous nuclear ribonucleoprotein K (hnRNP K)-targeted small interfering RNA (siRNA) on the growth and apoptosis of lung cancer cells *in vitro*. The expression of hnRNP K was studied by the SP method of immunohistochemistry in lung tissue samples of 70 cases of lung cancer. hnRNP K siRNA were transfected into human lung cancer cell line, A549, using Lipofectamine 2000. Cells transfected with siRNAn and untreated served as controls. The inhibitory effect of siRNA on the expression of hnRNP K mRNA and protein was detected by reverse transcription polymerase chain reaction and western blot analysis. The change in cell cycling and cell apoptosis of siRNA-treated cells was detected by flow cytometry. The rates of positive hnRNP K expression in lung tumors of diameters ≤3, 3–5 and ≥5 cm, were 38.5, 95.2 and 91.7%, respectively. A significant difference was identified between lung tumors with diameters of ≤3 and ≥3 cm (P<0.01). The expression of hnRNP K mRNA was significantly inhibited in siRNA-transfected cells compared with that in control cells (P<0.05). Notably, hnRNP K protein decreased in hnRNP K siRNA-transfected cells, but exhibited no effect on the control groups. siRNA targeting human hnRNP K effectively inhibited the growth of lung cancer cell line, A549, and the distribution of the cell cycle. The apoptosis rate was 4.79% and the number of cells increased in the G0/G1 phase from 37.21 to 85.60% and decreased in the S and G2/M phases from 47.71 to 13.50% and 14.00 to 0.32%, respectively, following 24 h of transfection. hnRNP K siRNA promotes A549 apoptosis and the apoptosis rate was 4.79% (P<0.01). Therefore, hnRNP K siRNA may inhibit the proliferation of A549 cells. In addition, hnRNP K promotes the growth of lung cancer cells and, therefore, hnRNP K siRNA may inhibit the growth and increase the apoptosis of lung cancer cells.

## Introduction

Heterogeneous nuclear RNAs (hnRNAs), from which mRNAs are generated by RNA processing, associate with specific nuclear proteins to form large hnRNP complexes (1,2). These hnRNP proteins bind pre-mRNAs and are considered to be important in mRNA biogenesis (3,4), nucleocytoplasmic transport of mRNA (5–7) and cytoplasmic mRNA trafficking (8). To date, 21 hnRNPs (A through U) have been identified. Certain family members are emerging as having an important involvement in tumor development (9).

Heterogeneous ribonucleoprotein K (hnRNP K) is a 464-amino acid protein with three K homology domains that mediate DNA and RNA binding and contain nuclear localization and nuclear shuttling domains (10,11). Based on these domains, we presumed that the hnRNP K protein is involved in multiple steps of gene expression, including transcription, RNA splicing and translation; such presumptions were later confirmed. In addition, a hnRNP protein potentially relevant in tumorigenesis is hnRNP K. In the nucleus, this protein binds directly to the promoter region of the human c-myc gene and functions as a transcription factor (12). When localized to the cytoplasm, hnRNP K inhibits the translation of specific mRNAs, such as 15-lipoxygenase mRNA (13). In breast cancer cells, hnRNP K significantly enhances cell proliferation and anchorage-independent growth through a growth factor dependent mechanism (14). The present study investigated the effects of hnRNP K siRNA on the growth of lung cancer cells *in vitro*.

## Materials and methods

### Study sample

A total of 70 cases of lung cancer were identified by pathology at the Department of Thoracic Surgery, West China Hospital of Sichuan University (Chengdu, China) between 2004 and 2005, in which there were 53 males and 17 females. The average age was 58.12 years and ranged between 46.2 and 70.04 years. The tumors from surgical resection included 13 samples with diameters of ≤3 cm, 20 samples with diameters between 3 and 5 cm and 56 samples with diameters of ≥5 cm. A549 lung cancer cell strains were obtained from the West China Hospital Respiratory Lab (Chengdu, China). Institutional review board approval for the present study was obtained from the Ethics Committee of Sichuan University (Chengdu, China) and written informed consent was obtained from all patients.

### Materials

Dulbecco’s modified Eagle’s medium, Lipofectamine™ 2000, TRIzol reagent and RPMI-1640 were purchased from Invitrogen Life Technologies (Carlsbad, CA, USA). The T7 RiboMAX™ Express RNAi system kit and Access reverse transctiption (RT)-polymerase chain reaction (PCR) introductory system were purchased from Promega Corporation (Madison, WI, USA). Monoclonal antibodies against hnRNP K were purchased from Santa Cruz Biotechnology, Inc. (Santa Cruz, CA, USA).

### Immunohistochemistry

Tumor tissues were fixed with paraformaldehyde (4%), paraffin-embedded and sectioned onto Plus slides (Thermo Electron Corp., Madison, WI, USA). Following antigen retrieval, endogenous peroxidase activity was inhibited (with 3% H_2_O_2_ in 50% methanol) and sections were blocked. Sections were then incubated with primary antibody. Following washing with phosphate-buffered saline (PBS), the sections were incubated with goat anti-mouse antibody conjugated to biotin. Then, following avidin-biotin-horseradish peroxidase amplification (Vectastain ABC Reagent; Vector Laboratories, Inc., Burlingame, CA, USA), the sections were incubated with filtered 3,3′-diaminobenzidine until the desired stain intensity had developed. Following subsequent washing with PBS, the slides were counterstained with hematoxylin. According to the Matthew classified criteria and combining the chromogenic strength and proportion of positive cells, the results were classified into three degrees between - and ++.

### Preparation of siRNA

The sequence data of human hnRNP K mRNA (BC025321; gi: 19116261) used were collected from GenBank (Bethsda, MD, USA). siRNA targeting human hnRNP K and one nonsense siRNA were designed online and obtained by transcription using a kit purchased from the Shanghai Shen Gong Chemical Co., Ltd. (Shanghai, China). The following sequences of hnRNP K siRNA were used: Sense: 5′-gatccccCTATTCCCAAAGATTTGGCttcaagagaGCCAAATCTTTGGGAATAGtttttggaaa-3′, anti-sense: 5′-agcttttccaaaaaCTATTCCCGATTTGGCtctcttgaaGCCAAATCTTTGGGAATAGggg-3′. The basic GC composition of the following nonsense siRNA was the same as for the siRNA targeting human hnRNP K: Sense, 5′-gatcccTATGGCGTACGTTATAATttcaagagaATTATCAACGTACGCCATAtttttggaaa-3′ and antisense: 5′-agcttttccaaaaaTATGGCGTACGTTGATAATtctcttgaaATTATCAACGTACGCCATAggg-3′, but had no distinguished homology with human hnRNP K RNA and was used as a negative control.

### Cell culture

The A549 cell line was maintained in RPMI-1640 supplemented with 10% fetal calf serum and incubated at 37°C in a humidified incubator containing 5% CO_2_.

### Cell cycle and apoptosis assay

Cells (1.5×10^5^/l) were suspended in RPMI-1640 and then plated onto 25-cm culture flasks. After gene transferring for 24 h, cells were collected, suspended in 0.01 mol/l PBS and fixed in 70% ethanol for 24 h. Cells were washed once with PBS, digested by RNase A (60 μg/ml) at 37°C for 30 min and stained with 1 ml propidium iodide (50 μg/ml) at 4°C for 30 min. DNA histograms were assayed by flow cytometry. In each sample, a minimum of 2.5×10^5^ cells were counted and stored in list mode. Data analysis was performed using standard CellQuest software (Becton-Dickinson, Franklin Lakes, NJ, USA).

### Semi-quantitative RT-PCR analysis

The following sequences of PCR primers were used for hnRNP K amplification: 5′-ctgcttcagagcaagaatgct-3′ and 5′-aactgcaggccctcttcca-3′. The predicted size of the PCR products was 200 bp. GAPDH served as a positive control. The following sequences of PCR primers were used for GAPDH amplification: are 5′-cctcaagatcatcagcaat-3′ and 5′-ccatccacagtcttctgggt-3′. The predominant cDNA amplification product was 141 bp in length. Cells (1.5×10^5^/l) were suspended in RPMI-1640, plated onto six-well culture plates and incubated in a humidified incubator containing 5% CO_2_ for 24 h at 37°C. Following transfection with siRNAs for 24 h, cells were collected and total RNA was extracted.

In total, 35 cycles of PCR were performed at 94°C for 3 min, 53°C for 30 sec, 72°C for 60 sec and a final extension at 72°C for 5 min. Following electrophoresis and ethidium bromide staining, DNA bands were visualized using an ultraviolet transilluminator (Ultra-Violet Products Ltd., Cambridge, UK). The results were scanned onto a computer to measure DNA band intensities.

### Western blot analysis

Cytoplasmic and nuclear protein fractions were prepared as previously described. For whole cell protein extraction, cells were lysed in 1 ml radioimmunoprecipitation assay lysis buffer. A total of 20 μg of protein was separated by 10% SDS-PAGE gel and subsequently transferred onto polyvinylidene fluoride membranes for western blot analysis. The following antibodies were used: Anti-hnRNP K (1:500); and HRP-conjugated secondary antibody (1:5,000).

### Determination of A549 cell growth and MTT viability

Each group of cells was transfected for 0, 24, 48, 72 and 96 h, to measure MTT absorbance and for cell growth curve mapping. The cell growth curve was produced with time (h) as the horizontal axis and the light absorption value as the vertical axis.

### Statistical analysis

Statistically significant differences were determined by one-way analysis of variance and the independent samples t-test. P<0.05 was considered to indicate a statistically significant difference.

## Results

### Expression of hnRNP K in lung cancer tumors

**Figure 1:**
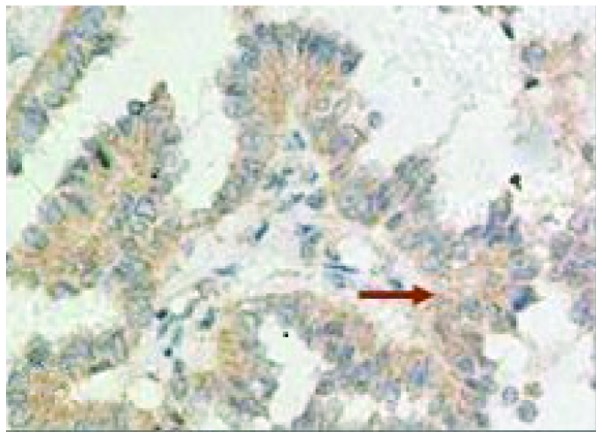
Immunohistochemical stainings of heterogeneous nuclear ribonucleoprotein K in lung cancer tumors (SP; magnification, ×200). Brown and yellow sections indicated by the arrow suggest positive staining.

The rates of positive hnRNP K expression in lung tumors with diameters of ≤3, 3–5 and ≥5 cm, were 38.5, 95.2 and 91.7%, respectively (P<0.01; Fig. 1 and Table I).

### Expression of hnRNP K in A549 cells

**Figure 2:**
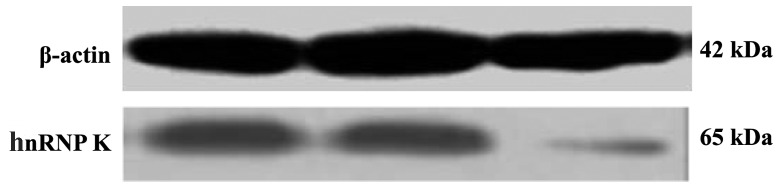
Western blot analysis of hnRNP K protein expression in different groups of A549 cells. hnRNP K, heterogeneous nuclear ribonucleoprotein K.

To examine the specific effect of hnRNP K siRNA treatment on hnRNP K expression in the A549 cell line, hnRNP K mRNA and protein expression levels were determined quantitatively using RT-PCR and western blot analyses, respectively (Fig. 2 and Table II). hnRNP K mRNA and protein were markedly expressed in lung cancer A549 cells as reflected by RT-PCR and western blot analysis. hnRNP K expression was decreased significantly at 24 h following transfection with specific hnRNP K siRNA.

### Effects of hnRNPK siRNA on cell cycle distributions and apoptosis rates

Compared with the hnRNPK siRNAn and untreated groups, specific hnRNP K siRNA caused an accumulation of cells in the G1 and S phases, decreased the number of cells in the G2/M phase and increased the hypodiploid DNA content (P<0.01) at 24 h following transfection (Table III). In the groups of specific hnRNP K siRNA, the apoptosis rate was 4.79% (Table III).

### hnRNP K siRNA on the proliferation of A549 cells

Following transfection at 0, 24, 48, 72 and 96 h, at each time point the cell growth activity of cells was determined. The results showed that compared with the control and siRNAn groups, the proliferation rate minimized (P<0.01) in the hnRNP K siRNA group of A549 cells at 48 h (Table IV).

## Discussion

The balance between the apoptosis and antiapoptosis signaling pathways is involved in the pathogenesis of a variety of cancers. It has been previously demonstrated that the inhibition of apoptosis promotes the mitotic progression in cancer cells (15).

hnRNP K protein is an abundant factor involved in transcription, mRNA processing and other events that compose gene expression. It is likely that the increased K protein levels observed in the nuclei of the proliferating cells serve to support nuclear processes that not only compose the inducible expression of a large number genes, but also maintains conducive chromatin topology in growing cells (16).

hnRNP K may promote cell proliferation and have a negative effect against the promotion of differentiation (17); in several states of enhanced cell proliferation, increased K protein levels have been identified in the nucleus. Induction of cell proliferation results in the activation of a large repertoire of genes (18). A previous study identified increased K protein expression in breast cancer cells. The authors provided evidence that the increased K protein levels contribute to the enhanced c-myc gene expression in such tumors (14).

In the present study, the expression and strength of hnRNP K was found to closely correlate with the size of tumor, specifically in the group of tumors with diameters of >3 cm, in which the positive rate of hnRNP K was >90%, a significant statistical difference compared with the group of tumors with diameters of ≤3 cm (the positive rate was 38.5%). This implied that hnRNP K may promote the growth and proliferation of lung cancer cells.

The c-Src interaction and activation domains in hnRNP K may be separated *in vitro* and in transfected cells. In the cellular context, the respective domains are present in hnRNP K. Therefore, the protein not only interacts with c-Src, as it does with lymphocyte-specific protein tyrosine kinase, but is also capable of activating c-Src in a specific manner. This supports the function of hnRNP K as a multifunctional scaffold protein that mediates the cross-talk between signaling pathways that controls cell differentiation and maturation (19). By interfering with the expression of hnRNP K in A549 lung cancer cell strains, the present study identified that the expression of the hnRNP K protein and mRNA decreased evidently following interference with hnRNP K siRNA compared with the non-interference group. This indicated that the hnRNP K gene is inhibited in transcription and at the protein expression level. The results obtained from flow cytometry indicated that hnRNP K siRNA inhibits the growth of A549 lung cancer cell strains, in which the cells remain in the G0/G1 stage. In addition, the increase of subdiploid DNA implied that hnRNP K siRNA induces cell apoptosis.

A major consequence of p53 activation following DNA damage is the induction of cell-cycle arrest at the G1/S or G2/M transition stages. This is achieved primarily through the p53-induced expression of target genes that encode factors, such as p21WAF/CIP, a negative regulator of cyclin-dependent kinases (CDKs) that induces G1/S arrest (20) and proteins, such as GADD45, 14-3-3s and Reprimo, which are required for an efficient G2/M arrest following DNA damage (21). The hnRNP K protein is required for p53-mediated transcription of cell cycle checkpoint genes (22). It enhances the transcription of oncogenes, such as c-myc and c-src and is considered to promote cell proliferation, survival and migration. hnRNP K has also been implicated in chromatin remodeling, mRNA splicing, export and translation (10). hnRNP K depletion abrogates the transcriptional induction of p53 target genes and causes defects in DNA damage-induced cell cycle checkpoint arrests. As a cofactor for p53, hnRNP K is key in coordinating transcriptional responses to DNA damage (22). hnRNP K is a multifunctional protein that has been studied primarily in cancer cells and has been suggested to be involved in cell cycle progression (23).

The results of the present study identified the significant increase of G0/G1 stage cells and significant decrease of G2/M stage cells in A549 lung cancer strains following transfection with hnRNP K siRNA. Previously, an inability of hnRNP K-depleted cells to induce p21, which normally mediates G1 arrest by inhibiting CDKs and by preventing the involvement of the proliferating cell nuclear antigen in DNA replication with DNA polymerase, has been identified (24,25). This result is consistent with the results of the current study, that the G0/G1 stage cells significantly increase in A549 strains following transfection with hnRNP K siRNA.

The present study supports that hnRNP K promotes the growth and proliferation of lung cancer cells and interfering with the hnRNP K expression may promote the apoptosis of lung cancer cells. Through the further investigation of the mechanism of hnRNP K promoting the growth and proliferation of lung cancer cells, new target sites of lung cancer therapy may be provided.

## Figures and Tables

**Table I:** Expression of hnRNP K in tumors of different diameters.

Tumor diameter, cm	n	hnRNP K expression			Positive rate, %

		−	+	++	
≤3	13	8	4	1	38.5
3–5	21	1	20	0	95.2^a^
≥5	36	3	25	8	91.7^a^

a P<0.01 vs. diameter of ≤3 cm.

hnRNP K, heterogeneous nuclear ribonucleoprotein K.

**Table II:** Expression of hnRNP K mRNA and protein in different groups of A549 cells.

Group	n	hnRNP K mRNA, 2^−ΔΔCt^	Expression ratio of hnRNP K proteins
hnRNP K siRNA	6	0.24±0.53^a^	0.23±0.12^a^
siRNAn	6	1.00	0.87±0.17
Controls	6	1.14±0.97	1.00±0.03

a P<0.01 vs. siRNAn and control groups.

hnRNP K, heterogeneous nuclear ribonucleoprotein K; siRNA, small interfering RNA.

**Table III:** Effects of treatment with hnRNP K siRNA on cell cycle distribution and apoptosis rate (%).

Group	Cell cycles			Apoptosis rate, %

	G0/G1	S	G2/M	
hnRNP K				
siRNA	85.60±3.94^a^	13.50±3.02^a^	0.32±0.07^a^	4.79±1.03^a^
siRNAn	42.53±2.78	43.28±4.12	14.20±2.90	1.05±0.43
Controls	37.21±3.39	47.71±2.73	13.00±0.92	0.86±0.14

a P<0.01 vs. siRNAn and control groups.

hnRNP K, heterogeneous nuclear ribonucleoprotein K; siRNA, small interfering RNA.

**Table IV:** Changes in the light absorption of cells in each group.

Time periods, h	hnRNP K siRNA	siRNAn	Controls
0	0.487±0.014^a^	0.499±0.035	0.501±0.004
24	0.193±0.003^b^	0.598±0.003	0.617±0.004
48	0.017±0.004^b^	0.690±0.011	0.720±0.004
72	0.30±0.007	0.739±0.011	0.751±0.015
96	0.504±0.004	0.768±0.011	0.776±0.007

a P>0.05 and

b P<0.01 vs. siRNAn and control groups.

hnRNP K, heterogeneous nuclear ribonucleoprotein K; siRNA, small interfering RNA.
